# Patient Use of Electronic Prescription Refill and Secure Messaging and Its Association With Undetectable HIV Viral Load: A Retrospective Cohort Study

**DOI:** 10.2196/jmir.6932

**Published:** 2017-02-15

**Authors:** D Keith McInnes, Stephanie L Shimada, Amanda M Midboe, Kim M Nazi, Shibei Zhao, Justina Wu, Casey M Garvey, Thomas K Houston

**Affiliations:** ^1^ Center for Healthcare Organization and Implementation Research Edith Nourse Rogers Memorial Veterans Hospital Bedford, MA United States; ^2^ Department of Health Law, Policy, and Management Boston University School of Public Health Boston, MA United States; ^3^ Division of Health Informatics and Implementation Science Department of Quantitative Health Sciences University of Massachusetts Medical School Worcester, MA United States; ^4^ Center for Innovation to Implementation VA Palo Alto Health Care System Palo Alto, CA United States; ^5^ Veterans and Consumers Health Informatics Office Office of Connected Care Veterans Health Administration Washington, DC United States; ^6^ School of Nursing Bouve College of Health Sciences Northeastern University Boston, MA United States

**Keywords:** health records, personal, HIV, viral load, electronic prescribing, electronic mail, secure messaging, self care, veterans

## Abstract

**Background:**

Electronic personal health records (PHRs) can support patient self-management of chronic conditions. Managing human immunodeficiency virus (HIV) viral load, through taking antiretroviral therapy (ART) is crucial to long term survival of persons with HIV. Many persons with HIV have difficulty adhering to their ART over long periods of time. PHRs contribute to chronic disease self-care and may help persons with HIV remain adherent to ART. Proportionally veterans with HIV are among the most active users of the US Department of Veterans Affairs (VA) PHR, called My HealtheVet. Little is known about whether the use of the PHR is associated with improved HIV outcomes in this population.

**Objective:**

The objective of this study was to investigate whether there are associations between the use of PHR tools (electronic prescription refill and secure messaging [SM] with providers) and HIV viral load in US veterans.

**Methods:**

We conducted a retrospective cohort study using data from the VA’s electronic health record (EHR) and the PHR. We identified veterans in VA care from 2009-2012 who had HIV and who used the PHR. We examined which ones had achieved the positive outcome of suppressed HIV viral load, and whether achievement of this outcome was associated with electronic prescription refill or SM. From 18,913 veterans with HIV, there were 3374 who both had a detectable viral load in 2009 and who had had a follow-up viral load test in 2012. To assess relationships between electronic prescription refill and viral control, and SM and viral control, we fit a series of multivariable generalized estimating equation models, accounting for clustering in VA facilities. We adjusted for patient demographic and clinical characteristics associated with portal use. In the initial models, the predictor variables were included in dichotomous format. Subsequently, to evaluate a potential dose-effect, the predictor variables were included as ordinal variables.

**Results:**

Among our sample of 3374 veterans with HIV who received VA care from 2009-2012, those who had transitioned from detectable HIV viral load in 2009 to undetectable viral load in 2012 tended to be older (*P*=.004), more likely to be white (*P*<.001), and less likely to have a substance use disorder, problem alcohol use, or psychosis (*P*=.006, *P*=.03, *P*=.004, respectively). There was a statistically significant positive association between use of electronic prescription refill and change in HIV viral load status from 2009-2012, from detectable to undetectable (OR 1.36, CI 1.11-1.66). There was a similar association between SM use and viral load status, but without achieving statistical significance (OR 1.28, CI 0.89-1.85). Analyses did not demonstrate a dose-response of prescription refill or SM use for change in viral load.

**Conclusions:**

PHR use, specifically use of electronic prescription refill, was associated with greater control of HIV. Additional studies are needed to understand the mechanisms by which this may be occurring.

## Introduction

Electronic health records (EHRs) are increasingly being adopted by hospitals, health plans, and other health care providers to improve the efficiency and effectiveness of health care delivery, meet provisions of the Affordable Care Act, and to qualify for Meaningful Use financial incentives [1-3]. Many of these EHRs include personal health records (PHRs, also known as “patient portals”), which enable patients to view parts of their medical record such as laboratory results, past and future appointments, and upcoming preventive care. Patients can also use PHRs to manage their own care and communicate with their health care providers including sending secure electronic messages to their clinical providers. A common PHR feature, electronic refill of prescriptions (hereafter called “Rx refill”), for example, may contribute to better medication adherence by ensuring that patients consistently have their medications on hand [4]. Similarly, there is evidence that secure messaging (SM) may contribute to increased likelihood of achieving chronic illness control, such as diabetes and hypertension, because of improved patient-provider communication [5-7]. These PHR self-management tools may be especially beneficial for persons with human immunodeficiency virus (HIV) who must carefully adhere to their combination antiretroviral therapy (ART), but also often have other chronic conditions that they must manage simultaneously [8].

Controlling the amount of HIV virus in the bloodstream is crucial to persons with HIV, and combination ART is highly effective at reducing HIV viral load when taken as prescribed. But the management of HIV is complex. Patients must carefully adhere to their ART regimen both to control viremia and to reduce the likelihood of drug resistance. Lab work is needed regularly to monitor HIV virus levels and the status of the immune system through CD4 cell counts [9]. Additionally, many persons with HIV have comorbid chronic mental and physical health conditions that may lead to drug-drug interactions or disease-drug interactions with ART [8]. This suggests that patients with HIV would benefit from frequent provider-patient communication and the ability to easily manage and order their medications [10-12]. PHR self-management tools, in particular Web-based prescription refills and SM, may facilitate patients’ adherence to ART and thus contribute to subsequent undetectable viral load [13,14]. However, to our knowledge no studies have examined whether the use of specific PHR features of prescription refill and SM are associated with improved clinical outcomes for persons with HIV.

This study examines use of the My HealtheVet PHR Rx refill and SM features by persons with HIV in a large integrated health care system. We sought to examine whether use of these tools was associated with undetectable viral load. To this end, we identified a cohort of HIV-infected veterans receiving US Department of Veterans Affairs (VA) health care and examined their patterns of My HealtheVet PHR use along with their laboratory results for HIV viral load status in 2 time periods.

## Methods

### Study Design

We conducted a retrospective cohort study, identifying veterans with HIV who used the My HealtheVet PHR and followed them to assess outcomes of suppression of viral load. The study was approved by the Bedford Massachusetts VA Medical Center Institutional Review Board.

### Setting

We used data from the VA system of medical records available through the VA Corporate Data Warehouse (CDW). Variables included patient demographics and International Classification of Disease, 9^th^ revision, clinical modification (ICD-9-CM) diagnosis codes associated with all VA inpatient and outpatient encounters from October 1, 2007, to March 31, 2012. These data were linked at the patient level with My HealtheVet registration, SM, and Rx refill data from April 2012.

### Sample

**Figure 1:**
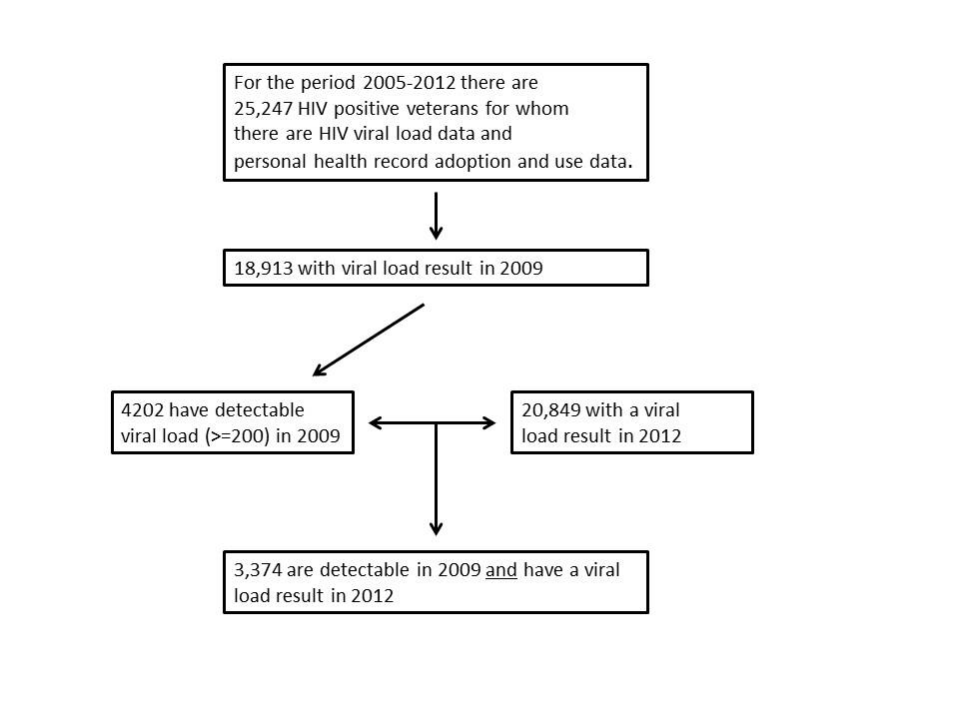
Creation of analytical database.

The study population included all American veterans aged 18 years and older who had obtained care from the VA health care system between April 1, 2010, and March 31, 2012 (N=6,012,875). Obtaining care in the VA was defined as having at least two outpatient visits or 1 inpatient hospitalization for any cause during this period. The cohort of HIV-infected patients was identified by examining VA’s decision support system laboratory results contained in CDW. Inclusion criteria for the cohort were: (1) a veteran determined to be HIV positive based on 2 or more instances of ICD-9-CM codes for HIV in the CDW, (2) had a detectable viral load in 2009, and (3) had viral load test results (detectable or undetectable) in 2012. For these analyses, an undetectable viral load was considered less than 200 copies of the HIV virus per milliliter (mL) of blood (<200/mL); conversely, 200 copies or more of the HIV virus per mL of blood (>=200/mL) indicated a detectable viral load. We identified 18,913 veterans who were HIV-positive and had a viral load test result in 2009 (See Figure 1). Of these, 4202 had a detectable viral load. Among these 4202 there were 3374 veterans who had a viral load test result in 2012. These 3374 veterans were the subject of this study. However, in the multivariable analyses, the total sample was 3289 due to missing values for 85 patients. For patients with multiple viral load tests in a given year, the most recent result in that calendar year (closest to December 31) was used to categorize the patient’s viral load status.

### VA’s Personal Health Record, My HealtheVet

VA launched the My HealtheVet PHR in 2003 and enrollment has grown to approximately 3.5 million current registered users as of January 2016 [15]. The PHR provides evidence-based health information, health logs (eg, to track diet, exercise, weight), access to providers’ notes, reminders for preventive health services (eg, immunization, cancer screening), prescription drug refills, and secure email messaging [16]. Since 2005, over 70 million prescription refills have been requested on the Web, and 1.7 million veterans have signed up for the SM service. Interestingly, veterans with HIV register for the My HealtheVet PHR at higher rates than veterans with other chronic illnesses. While 18.64% of all veterans receiving treatment from VA were registered to use the PHR in 2012, 26.48% of veterans with HIV were registered at the time [15].

### Data Collection

#### Independent Variables

Use of My HealtheVet Rx refill and SM features were the independent variables of interest. We examined whether each of these tools was used by each veteran in our sample in the 3-year follow-up period from 2010-2012, and whether use of the tool was associated with undetectable viral load. These 2 variables were dichotomous with 1 indicating use of Rx refill at least one time in the 2010-12 period, and 0 indicating no use of Rx refill during that time period. The SM variable was created in the same way, with 1 indicating at least one use in 2010-12 and 0 indicating no use during that time period. In separate analyses, we also explored whether there may be a dose-response such that sustained use of the tool (Rx refill or SM) was associated with an increased likelihood of having an undetectable viral load. For those analyses, each of the variables (Rx refill and SM) was coded as an ordinal variable with 0 indicating no use in 2010-12, 1 indicating use in only 1 of the 3 years, and 2 indicating use in 2 or more of the 3 years.

#### Dependent Variable

The outcome of interest was viral load status in 2012. It was coded 1 for viral load < 200 mL (undetectable) and 0 for viral load >=200 mL (detectable).

#### Covariates

Following Shimada et al who examined electronic prescription refill and SM among 6 million veterans using VA health services, we included in our multivariable analyses the following demographic characteristics: age, gender, race or ethnicity, urban or rural residence based on home postal code, and economic need defined as eligibility for free care based on an annual VA means testing [15]. We grouped participants by age (<45 years or 45-54 years or 55-64 years or 65+ years), marital status (married vs never married or divorced or separated or widowed), and race or ethnicity (African American vs White or Asian or Native Hawaiian or American Indian or other). Chronic conditions and disease burden were measured using ICD-9-CM medical and mental health diagnoses that appeared at least once for an inpatient stay or at least twice for outpatient visits. The diagnostic grouping to identify chronic conditions had been previously validated in a large VA longitudinal study of veterans with HIV [17]. Comorbidities may have influenced both disease self-management behaviors and interest in using the PHR. We included comorbidities in our models in 2 ways. First, problem alcohol use, other substance use, depression, and psychoses were each included as independent variables, following their identification using ICD-9-CM codes, as described earlier. These 4 conditions were singled out because of their relatively high prevalence among HIV-infected populations [18,19] and their known negative association with medication adherence [20-22]. In addition, as an overall measure of chronic disease burden, we used the Elixhauser score [23-25]. This is a summary measure of disease burden that combines 31 common comorbidities. Because all patients in our sample were HIV positive, we adjusted the Elixhauser measure by removing HIV disease. In addition, we removed problem alcohol use, substance use, depression, and psychoses from the Elixhauser measure because they had already been included as independent variables as described previously.

### Analyses

We began by examining bivariate relationships between HIV viral control and the independent variables using cross-tabulation and chi-square tests. To assess relationships between Rx refill and viral control, and SM and viral control, with the sample of 3289 patients, we fit a series of multivariable generalized estimating equation models, accounting for clustering in VA facilities. To understand how patient characteristics might bias the primary association of Rx refill (or SM) and viral control, our models controlled for socio-demographics (age, gender, race or ethnicity, economic need, marital status, rural or urban status) and health status (ie, Elixhauser comorbidity burden, as well as separate indicators of alcohol use, other substance use, depression, and psychoses). We ran models for Rx refill and SM separately. In initial models the predictor variables were included in dichotomous format. Subsequently, to evaluate a potential dose-effect, the predictor variables were included as ordinal, as described previously. This produced 4 different models. We considered statistical significance to be *P*<.05. To assess potential multicollinearity, we estimated variance inflation factor (VIF) for each independent variable in each of our multivariable models. No variables had VIF greater than 10 (the largest VIF was 1.61) suggesting there was little or no multicollinearity. This also suggests sampling bias was not a substantial issue. To assess whether being registered to use the PHR or not would affect our findings, we conducted sensitivity analyses in which we restricted the sample to the 1130 registered patients. When we ran the same 4 regression models, our results did not differ substantially from the original 4 models. Thus in the results section discussed later on, we focus on the analyses using the larger sample of 3289 patients.

## Results

### Patient Characteristics and PHR Use

Sociodemographic, health, and PHR use data are shown in Table 1, by viral load detectable status in 2012. Of the 3374 who had detectable viral loads in 2009, 66.60% had undetectable viral loads in 2012. Nearly three-quarters (71.61%) of the sample were in the 45-64 year age range, and 96.21% were male (consistent with the demographics of the US veteran population). The majority of the sample was African American (61.53%). Close to half (46.06%) were considered having high economic need, based on eligibility for free VA health care services due to low income, and 11.47% were married. Most of the sample (88.97%) lived in urban areas. There were considerable mental health and substance use disorder morbidity, including 60.04% with a diagnosis of depression, 41.44% with a substance use disorder, 32.99% with problem alcohol use, and 6.32% with psychoses. Excluding the aforementioned 4 diagnoses and HIV, subjects had a mean of 2.7 chronic conditions (as assessed with the Elixhauser score). One-third (33.49%) had registered for the PHR as of 2012. In addition 17.81% of the sample had used the PHR’s electronic prescription refill at least once in the 2010-2012 period, and 5.93% had sent at least one secure message to a VA provider during that period.

**Table 1:** Participant characteristics, overall and by human immunodeficiency virus (HIV) viral control in 2012.

Variable		Overall, n (%)	Viral control		*P* value
			Viral load undetectable, n (%)	Viral load detectable, n (%)	
		3374 (100)	2247 (66.60)	1127 (33.40)	
**Age in years**					.003
	<45	637 (18.88)	414 (18.42)	223 (19.79)	
	45-54	1190 (35.27)	776 (34.53)	414 (36.73)	
	55-64	1226 (36.34)	814 (36.23)	412 (36.56)	
	65+	321 (9.51)	243 (10.81)	78 (6.92)	
**Gender**					.05
	Female	128 (3.79)	75 (3.34)	53 (4.70)	
	Male	3246 (96.21)	2172 (96.66)	1074 (95.30)	
**Race**					<.001
	White	1130 (33.49)	826 (36.76)	304 (26.97)	
	Black	2076 (61.53)	1309 (58.26)	767 (68.06)	
	Hispanic	28 (0.83)	15 (0.67)	13 (1.15)	
	Native Hawaiian	29 (0.86)	24 (1.07)	5 (0.44)	
	American Indian	14 (0.41)	10 (0.45)	4 (0.35)	
	Asian	11 (0.33)	8 (0.36)	3 (0.27)	
	Unknown	86 (2.55)	55 (2.45)	31 (2.75)	
**Economic need (means test)**					.22
	Eligible for free care	1554 (46.06)	1018 (45.30)	536 (47.56)	
	Not eligible	1820 (53.94)	1229 (54.70)	591 (52.44)	
**Marital status**					.001
	Married	387 (11.47)	259 (11.53)	128 (11.36)	
	Never married	1567 (46.44)	1040 (46.28)	527 (46.76)	
	Divorced	1037 (30.74)	700 (31.15)	337 (29.90)	
	Separated	228 (6.76)	154 (6.85)	74 (6.57)	
	Widowed	120 (3.56)	83 (3.69)	37 (3.28)	
	Others	35 (1.04)	11 (0.49)	24 (2.13)	
**Urban or rural location**					.18
	Missing	82 (2.43)	51 (2.27)	31 (2.75)	
	Urban	3002 (88.97)	1990 (88.56)	1012 (89.80)	
	Rural	290 (8.60)	206 (9.17)	84 (7.45)	
**Comorbidities**					
	Depression (% yes)	2024 (60.04)	1325 (58.97)	699 (62.19)	.07
	Substance use (% yes)	1397 (41.44)	894 (39.79)	503 (44.75)	.006
	Problem alcohol use (% yes)	1112 (32.99)	713 (31.73)	399 (35.50)	.03
	Psychoses (% yes)	213 (6.32)	123 (5.47)	90 (8.01)	.004
	Elixhauser comorbidity score, mean (STD)	2.69 (2.35)	2.67 (2.38)	2.72 (2.31)	.57
**My HealtheVet PHR Use**					
	Registered to use My HealtheVet as of 2012	1130 (33.49)	785 (34.94)	345 (30.61)	.01
	Use Rx refill (2010-2012)	601 (17.81)	435 (19.36))	166 (14.73)	<.001
	Use secure messaging (2010-2012)	200 (5.93)	147 (6.54)	53 (4.70)	.03

There were differences between veterans with undetectable versus detectable viral load. Veterans with undetectable viral load were older (10.81% vs 6.92%, 65+ years), more likely to be male (96.66% vs 95.30%), white (36.76% vs 26.97%), and divorced (31.15% vs 29.90%). They were less likely than veterans with detectable viral load to have symptoms of depression (58.97% vs 62.19%), substance use disorder (39.79% vs 44.75%), problem alcohol use (31.73% vs 35.50%), or psychoses (5.47% vs 8.01%). Those with undetectable viral load were also more likely to be registered for My HealtheVet (34.94% vs 30.61%), to have used Rx refill (19.36% vs 14.73%), and to have used SM (6.54% vs 4.70%).

### Multivariable Models

In our multivariable model examining use of Rx refill, there was a positive association between use of Rx refill and viral load control (Table 2). Veterans using Rx refill had 1.36 the odds (95% CI 1.11-1.66) of having an undetectable viral load compared with veterans who did not use Rx refill, after adjusting for sociodemographic and health variables. In addition, older age (Odds ratio, OR 1.01, 95% CI 1.00-1.02) and being white (OR 1.49, 95% CI 1.21-1.83) were independently and positively associated with undetectable viral load, whereas a diagnosis of psychosis (OR 0.66, 95% CI 0.46-0.95) was associated with reduced likelihood of undetectable viral load.

**Table 2:** Multivariable analysis of the odds of undetectable viral load in 2012 in relation to Rx refill use (n=3289).

Variable	Estimate	Odds ratio	95% CI	*P* value
Use Rx refill 2010-2012 (dichotomous)	0.3085	1.36	1.11-1.66	.003
Age	0.0132	1.01	1.00-1.02	.004
Male	0.2555	1.29	0.86-1.94	.22
White race	0.399	1.49	1.21-1.83	<.001
Economic need (means test)	-0.101	0.90	0.78-1.05	.19
Married	-0.053	0.95	0.78-1.16	.61
Elixhauser score	-0.009	0.99	0.96-1.03	.61
Problem alcohol use	-0.007	0.99	0.84-1.17	.93
Substance use	-0.059	0.94	0.76-1.18	.60
Depression	-0.067	0.94	0.79-1.11	.44
Psychoses	-0.417	0.66	0.46-0.95	.03
Rural	0.1506	1.16	0.86-1.57	.33

There were similar associations when viral load was modeled with SM as the predictor variable. However, SM did not achieve a statistically significant association with viral load status (Table 3). Veteran using SM had 1.28 the odds (95% CI 0.89-1.85) of having undetectable viral load compared with veterans who did not use SM, after adjusting for sociodemographic and health variables. Similar to the Rx refill model, older age (OR 1.01, 95% CI 1.00-1.02) and being white (OR 1.53, 95% CI 1.25-1.86) were independently and positively associated with undetectable viral load. Conversely, a diagnosis of psychosis (OR 0.66, 95% CI 0.46-0.95) was associated with reduced likelihood of having an undetectable viral load.

**Table 3:** Multivariable analysis of the odds of undetectable human immunodeficiency virus (HIV) viral load in 2012 in relation to secure messaging (SM) use (n=3289).

Variable	Estimate	Odds ratio	95% CI	*P* value
Use secure messaging	0.2462	1.279	0.89-1.85	.19
Age	0.0114	1.012	1.00-1.02	.01
Male	0.2748	1.316	0.87-1.99	.19
White race	0.4231	1.527	1.25-1.86	<.001
Economic need (means test)	-0.107	0.898	0.78-1.04	.15
Married	-0.063	0.939	0.77-1.15	.53
Elixhauser score	-0.008	0.992	0.96-1.03	.64
Problem alcohol use	-0.017	0.983	0.84-1.16	.83
Substance use	-0.073	0.929	0.75-1.16	.51
Depression	-0.057	0.944	0.80-1.12	.51
Psychoses	-0.416	0.659	0.46-0.95	.03
Rural	0.1509	1.163	0.86-1.58	.33

There was no evidence of a dose effect for either Rx refill or SM when treated as ordinal variables (Tables 4 and 5). The overall association of Rx refill with undetectable viral load, however, was still evident. Compared with veterans with 0 uses of Rx refill, those with Rx refill use in 1 year had 1.38 the odds of undetectable viral load (95% CI 1.02-1.87) and those with Rx refill use in 2+ years had nearly the same odds ratio (OR 1.35, 95% CI 1.07-1.71) (Table 4).

**Table 4:** Multivariable analysis of the odds of undetectable human immunodeficiency virus (HIV) viral load in 2012 in relation to Rx refill use (Rx refill included as ordinal variable), for assessment of dose effect (n=3289).

Variable	Estimate	Odds ratio	95% CI	*P* value
Use Rx refill 0 years (ref)	-	-	-	-
Use Rx refill 1 year	0.3194	1.376	1.02-1.87	.04
Use Rx refill 2-3 years	0.3016	1.352	1.07-1.71	.01
Age	0.0132	1.013	1.00-1.02	.004
Male	0.2556	1.291	0.86-1.94	.22
White race	0.3995	1.491	1.21-1.83	<.001
Economic need (means test)	-0.101	0.904	0.78-1.05	.19
Married	-0.052	0.949	0.78-1.16	.61
Elixhauser score	-0.009	0.991	0.96-1.03	.61
Problem alcohol use	-0.007	0.993	0.84-1.17	.93
Substance use	-0.059	0.943	0.76-1.17	.60
Depression	-0.067	0.935	0.79-1.11	.44
Psychoses	-0.417	0.659	0.46-0.95	.03
Rural	0.1501	1.162	0.86-1.57	.33

The findings for SM also indicated no evidence of dose effect. The odds ratio for SM use in 1 year was 1.37 (95% CI 0.88-2.13) and for SM use in 2+ years was 1.02 (95% CI 0.51-2.01) though neither was statistically significant (Table 5).

**Table 5:** Multivariable analysis of the odds of undetectable human immunodeficiency virus (HIV) viral load in 2012 in relation to secure messaging (SM) use (as ordinal variable), for assessment of dose effect (n=3289).

Variable	Estimate	Odds ratio	95% CI	*P* value
Secure messaging 0 years (reference)	-	-	-	-
Secure messaging 1 year	0.3157	1.371	0.88-2.13	.16
Secure messaging 2-3 years	0.0149	1.015	0.51-2.01	.97
Age	0.0114	1.012	1.00-1.02	.01
Male	0.2759	1.318	0.87-1.99	.19
White race	0.4252	1.53	1.25-1.87	<.001
Economic need (means test)	-0.106	0.9	0.77-1.04	.16
Married	-0.064	0.938	0.77-1.14	.53
Elixhauser score	-0.008	0.992	0.96-1.03	.62
Problem alcohol use	-0.017	0.983	0.84-1.16	.84
Substance use	-0.073	0.929	0.75-1.16	.51
Depression	-0.056	0.945	0.80-1.12	.51
Psychoses	-0.421	0.657	0.45-0.95	.03
Rural	0.1501	1.162	0.86-1.58	.34

Our sensitivity analyses, in which the regression models were reestimated using the restricted sample of the 1130 registered patients, did not yield any substantively different results than those presented in Tables 2-5- (data not shown).

## Discussion

### Principal Findings

We found that among veterans with HIV, there was a positive association between My HealtheVet PHR use and undetectable viral load. Specifically, veterans who had detectable viral loads in 2009, and who used the Rx refill function between 2010 and 2012, had 1.36 times the likelihood of undetectable viral load in 2012 compared with veterans who did not use Rx refill. There was no evidence of dose effect of either of Rx refill or SM on the likelihood of undetectable viral load. No observational study can prove causality, but we had features that support a causal argument—we identified a longitudinal relationship, but not a clear dose-response. Thus it is possible that PHR use is a marker for some unmeasured covariates, such as engagement with the health care system.

### Limitations

Due to the study limitations, we cannot rule out that there may be other explanations for our main finding of an association between use of Rx refill and undetectable viral load. The most salient limitation is that as an observational study there is potential for confounding by indication, in that patients who are already activated to improve their health may also be more likely to try new tools, such as PHRs. It is possible that more empowered and self-efficacious patients decide to use a PHR, whereas patients who are less motivated and more challenged with self-management tasks do not. Self-efficacy and empowerment may be driving forces behind achievement of undetectable viral load, and not actually PHR use alone. However, by limiting our analytic sample to the HIV positive veterans who had uncontrolled viral load in 2009, we sought to minimize variation in self-efficacy and empowerment. Another important variable, which was not available in our dataset and thus not included in our models, is stigma. Data indicate that Web-based tools are seen as particularly valuable, and used more often, among persons with stigmatized health conditions than among persons with nonstigmatized conditions [26]. Degree of perceived stigma may have been affecting the results found in our study.

Additionally, the linkage between Web-based prescription refill and undetectable viral load is presumably mediated by proper medication taking and medication adherence. Our data did not permit us to evaluate medication adherence, or whether the PHR prescription refills were specifically for ART as compared with medications for other comorbid conditions (eg, for diabetes, hypertension). Additional studies are needed that address these limitations, for example by randomizing patients to PHR use, and by assessing ART adherence to see whether the trajectory from PHR use to undetectable viral load occurs though the expected taking of ART. Our sample was all veterans and mostly male, therefore our results may not be generalizable to nonveterans and to women. Another way that generalizability may be limited is that the VA is unlike many smaller health care systems in that it has succeeded in registering a large number of its patients for PHR use, approximately 3.5 million users as of 2016 [15]. This number is likely only exceeded in the United States by the number of users in the Kaiser Permanente health care system [27]. Additionally the VA’s PHR has more features than many other PHR systems (including the recent addition of OpenNotes—that is, patient access to clinician notes [28]), which may be one of the reasons for the relatively high patient enrollment rates. While these unique features of the VA’s PHR reduce generalizability, alternatively the sheer number of users makes the VA an ideal environment for examining the impact of PHRs on health care delivery, processes, and outcomes, even for conditions with relatively low prevalence such as HIV.

### Comparison With Prior Work

Overall evidence on the effect of PHR use on care processes and outcomes for a variety of health conditions show mixed results [29]. While Zhou et al found an association between patient SM use and improved intermediate outcomes for hypertension, cholesterol, and diabetes [5], a clinical trial conducted by Wagner et al randomly assigning subjects to PHR access or no access found no effects of PHR access on hypertension control, though subanalyses of frequent PHR users showed they achieved reductions in diastolic blood pressure, compared with the infrequent PHR users [30]. In a retrospective study of patients with diabetes at the Cleveland Clinic, Tenforde et al found in adjusted multivariable analyses that in a PHR user group compared with nonuser group there was more hemoglobin A1c (HbA1c) testing and lower HbA1c values (7.0% in the PHR group vs 7.3% in the non-PHR group; *P*<.01) [31]. A study of veterans who were using the My HealtheVet PHR found that use of Rx refill and SM were both associated with improved low density lipoprotein cholesterol levels. It also found sustained use (use each year over 3 or more years) of SM was associated with improved HbA1c levels among veterans with diabetes, whereas sustained use of Rx refill was associated with improved blood pressure control among veterans with hypertension [32]. In yet another diabetes study conducted with 54 patients at Vanderbilt University Medical Center, Wade-Vuturo et al found that use of the PHR’s SM feature (more vs fewer messages sent) was moderately associated with lower HbA1c values in Spearman correlation analyses, (ρ=-.29, *P*=.04) [33].

In HIV, similarly, the evidence is mixed, with several studies suggesting that PHR use may assist with care processes and outcomes, but at least one study finding no association. A 6-site study by Shade et al [34] used a serial cross-sectional design in which each site selected a different health IT intervention to improve HIV care. One site introduced a continuity of care summary accessible to patients via a portal. At that site, based on data from 500 patients, there was an increased odds of undetectable viral load from baseline (prior to patient continuity of care summary) to follow up (OR 1.36, 95% CI 1.09-1.71) [34,35]. Patients were selected to be representative of the clinic population without regard for whether they used the continuity of care summary. A study by Crouch et al [36] found that PHR use was associated with improved HIV control. The team identified 80 HIV+ veterans using a nonprobability quota sample, selected to be split evenly between using and not using VA’s My HealtheVet PHR. There was a statistically significant association between My HealtheVet PHR use and undetectable viral load, with 95% having undetectable viral load in the PHR group compared with 70% in the non-PHR group (*P*=.046) [36]. This study did not examine which components of the PHR patients used. McInnes et al reported that in a cross-sectional sample of 1871 veterans, multivariable analyses found that self-reported PHR use was associated with pharmacy refill ART adherence [4]. The findings from logistic regression analyses by Gordon et al, in contrast to the other studies, found in comparing 39 PHR users with 43 nonusers that there was no association of PHR use with either self-reported ART adherence or medical record viral load control [37]. What the evidence in HIV and in other health conditions suggests is that there is greater need for randomized studies. This becomes more difficult as PHRs are increasingly included with EHR packages that hospitals and health systems adopt; however, there are still patient groups that have had little or no PHR exposure.

Our findings also point to potential racial disparities in access to and use of HIV related care. In each of our multivariable analyses, white patients had greater odds of achieving viral control than other races (which were overwhelmingly black). This may be due to uncontrolled variables such as education level which may vary by race. Other research, including studies in the VA health care system, suggests various sources for racial disparities. Richardson et al recently examined racial differences in HIV care (and in comorbid care for HIV-infected patients) in VA and reported, “Despite the lack of insurance-related barriers to care in the equal-access VHA health care system, racial disparities in the care for veterans with HIV remain problematic and extend to comorbid conditions” [38]. Whereas the knowledge about the causes of these disparities is incomplete, Richardson et al suggest that possible contributors include patient attitudes and beliefs, provider attitudes and implicit biases, differences in patient-provider communication, and social-structural disadvantages by race [39-42].

The persistent finding, in each of our models, of a negative association between the presence of psychoses and uncontrolled viral load is noteworthy. This may indicate patients in this group have difficulty adhering to their HIV medications. Research into nonadherence (in general, not just for HIV) for persons with psychoses suggests that there are a number of potential barriers to adherence to medications. These include lack of social support, problems with therapeutic alliance, lack of daily routines, negative attitudes toward medications, and cognitive deficits [43].

More broadly, among persons with HIV, adoption and use of PHRs have been associated with a number of sociodemographic characteristics. A study of veterans with HIV found that PHR use is associated with younger age, less than excellent or very good health, white race, more education, lack of substance use disorder, and higher incomes [4]. Patient PHR use also has some advantages over face-to-face communication. For health-related questions that are perceived by patients as sensitive or that may cause embarrassment or perceived stigma, PHRs provide a sense of privacy and anonymity [26,44].

PHRs provide patients with greater access to their providers and health information, and also provide tools that allow patients to undertake health self-management tasks more efficiently than through in-person or phone contact with their health care team [45-47]. Patients report that accessing information in their PHR can enhance communication with providers, for example through improved preparation for in-person visits. PHRs and the information they contain also aid patients by improving knowledge of their health, serving as reminders for needed follow up, and creating a sense of accountability for one’s health that leads to more self-care [48]. Accordingly, PHRs can be expected to enable patients to increase self-management of chronic conditions, resulting in better control of those conditions and better health overall. However, it is unclear whether simply providing a PHR to patients is sufficient to achieve this, as indicated by the results of the study by Wagner et al described previously [30]. With the provision of PHRs, there may be a need for patient orientation, training, and continued promotion to increase both adoption and sustained use, and to encourage specific uses that are likely to have the most health-related benefits.

### Conclusions

Our examination of PHR use adds important information to the existing body of work. Our data come from a highly stigmatized and vulnerable population of veterans with HIV, many of whom have high economic need, are racial or ethnic minorities, have a mental health or substance use disorder, and may lack social support (only 11% married). Interestingly, this population has been shown to use VA’s My HealtheVet PHR more than other chronic disease groups [15]; however, the cause of this phenomenon is not completely clear. That the use of a PHR electronic prescription refill feature was positively associated with undetectable viral load status in this vulnerable population is encouraging in that it may indicate an augmentation of access to medications and providers. The PHR tools of Rx refill and SM may afford patients a greater sense of freedom to perform functions—ordering refills and communicating with providers—unencumbered by the risk of social stigma associated with face-to-face or phone contacts [26]. Reducing such stigma may encourage greater patient interactions with providers and the health care system when issues arise, and improve self-efficacy in addressing health-related tasks and challenges. This in turn should help reduce the occurrence of patients running out of prescription medications and also facilitate swift resolution of serious side effects a patient may be experiencing from medications or drug-drug interactions.

Given our observational study design, however, further examination of the potential benefits of PHRs, and tools such as electronic prescription refill, are merited, especially if they can involve randomization. That a considerable proportion of this population is using PHR tools is encouraging—and yet about two-thirds do not. This suggests that continued efforts are needed to reach out to this population and to provide eHealth tools that are seen as easy to use and beneficial, regardless of the background or socio-economic and health status of the potential user.

## Abbreviations

ART: antiretroviral therapy
CDW: Corporate Data Warehouse
EHR: electronic health record
HbA1C: Hemoglobin A1c
HIV: human immunodeficiency virus
IT: information technology
mL: milliliter
OR: odds ratio
PHR: personal health record
SM: secure messaging
VA: Department of Veterans Affairs
